# Developing a career and education framework for advanced clinical practice in midwifery

**DOI:** 10.18332/ejm/188115

**Published:** 2024-05-24

**Authors:** Ruth Sanders, Katherine Letley, Kelda Folliard, Melanie Applegate, Kirsty Tweedie, Kenda Crozier

**Affiliations:** 1 Faculty of Medicine and Health Sciences, School of Health Sciences, University of East Anglia, Norwich, United Kingdom

**Keywords:** advanced clinical practice, midwifery, midwifery career, scope of practice, midwifery education, midwifery career progression

## Abstract

**INTRODUCTION:**

This study outlines the nature of Advanced Clinical Practice in Midwifery (ACPiM), reporting on a stakeholder analysis as part of a national project to develop a career framework for advanced practice in midwifery on behalf of the National Health Service (NHS) in England.

**METHODS:**

Between June and July 2022, 31 advanced practice midwives were recruited across England within the NHS settings. Convenience sampling was used to identify midwives working as advanced practitioners, and those pursuing this career route. Focus group and one-to-one interviews were conducted, recorded, and transcribed. These stakeholder data were then analyzed using a reflexive thematic approach.

**RESULTS:**

ACP midwives were active across many professional settings. The findings resulted in three themes: Midwifery autonomy, Desire for progression, and Avenues of support. Midwifery autonomy highlighted a midwifery desire to utilize specialist skills and expert decision-making to provide holistic care directly to women and families. Desire for progression highlighted that, regardless of career stage, midwives aspired to advance their practice requiring a range of pathways to fulfil career satisfaction and meet local population health needs. Avenues of support discussed the barriers and facilitators to progression, highlighting the need for service vision, a multi-disciplinary approach to facilitate support for individuals, and strong midwifery leadership.

**CONCLUSIONS:**

Although the ACPiM role is desired by maternity institutions and organizations, midwives remain unclear about how to achieve this position, and employers remain unsure of how an ACPiM could transform services. If midwives are to successfully achieve ACPiM status, organizational support is needed to facilitate individuals drive for career progression, resulting in a strengthened workforce and improved patient experience.

## INTRODUCTION

The role of the midwife has evolved, with changes reflected in the UK standards of proficiency for midwives which encompass a range of skills necessary for midwives at the point of registration^1^. As midwives move beyond preceptorship and the early years of their careers, many aim for enhanced roles, specializing in areas of interest such as diabetes or bereavement midwifery care. Midwives may aspire to advanced practice or consultant level practice, remaining close to clinical care and there should be the opportunity to pursue this career pathway^2,3^.

Consensus internationally on advanced clinical practice specific to midwifery is limited with a Belgian study^4^ describing a lack of cohesion among senior midwives about the roles that should be performed in advanced midwifery practice, because the number of advanced practice role titles is small, whilst there appears to be a growing range of clinical specialist roles^5^. In 2009, the Australian College of Midwives rejected advanced practice, stating it represented midwives exercising their existing full scope of practice. In the same year the Royal College of Midwives recognized advanced capabilities when it described four areas of responsibility for consultant midwives, i.e. expert clinical practice, clinical and professional leadership, research and education, and practice and service development^6^.

Irish studies^7-9^ described roles held by clinical specialist midwives, which encompassed elements of advanced practice such as education, research co-ordination and publishing as well as providing complex care. This body of evidence informed development of standards for ACPiM by National Board for Nursing and Midwifery, Ireland (NMBI 2018)^10^. The NMBI competencies include: advanced assessment and intervention strategies; research applied to making clinical decisions; analyzing complex interactions; guiding decision-making; and developing client focused care. The document recognizes the need for leadership and infrastructure to support these roles in organizations. The need for an educational pathway with support from higher education and employers is also highlighted to ensure sustainability.

The UK surveys conducted by Wilson et al.^[Bibr cit0011]11,[Bibr cit0012]12^ provided us with the largest source of data on advanced practice in UK midwifery prior to our work. The surveys identified 84 consultant midwife posts in the earlier study and 93 in the most recent. The articles are lacking in specific detail of the roles, responsibilities and educational preparation of those post holders. It appears that many of the roles are centered on leadership of birth centers. Roles appear to encompass leadership and service development with little information on education or research aspects.

Recent attention has been directed towards a clearer definition of advanced clinical practice in midwifery, with the publication of the Advanced Clinical Practice in Midwifery capability framework (ACPiM)^13^ (Health Education England, 2022) building this based on the UK multiprofessional framework:

*‘It is a level of practice characterized by a high degree of autonomy and complex decision making. This is underpinned by a Master's level award or equivalent that encompasses the four pillars of clinical practice, leadership and management, education, and research, with demonstration of core capabilities and area specific clinical competence. Advanced clinical practice embodies the ability to manage clinical care in partnership with individuals, families and carers. It includes the analysis and synthesis of complex problems across a range of settings, enabling innovative solutions to enhance people's experience and improve outcomes.’* (NHS HEE 2017)

Developed in collaboration with key stakeholders, this work illustrated a standardized level of practice for the future ACP midwife. In providing context to this article, it is key to outline the ACP in midwifery capability framework itself. The midwifery specific capabilities were formulated to complement the four pillars of advanced clinical practice^13^, having mapped them against the UK Nursing and Midwifery Council proficiencies for midwives^1^.

### The ideal ACP midwife

In critically examining the current state of ACP in midwifery, it is helpful to consider how the ideal ACP midwife would look and how individuals’ midwifery practice may be divided across the four pillars. As evidence suggests, clinical practitioners have limited time to read and access reports^14^, so an image format was used to enhance clarity. It was a critical aim for these findings to be shared and translated to maximize the benefit to our target audience of midwives and midwifery leaders.

**Figure 1:**
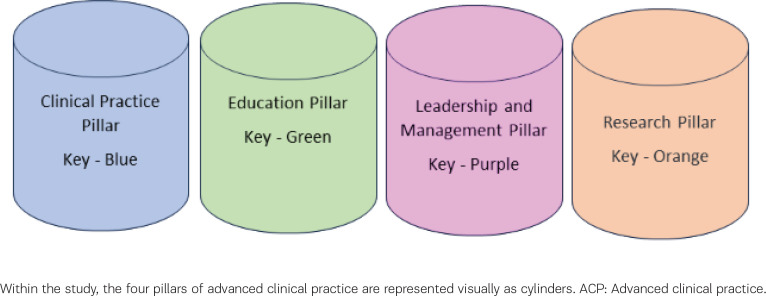
The four ACP pillars

**Figure 2:**
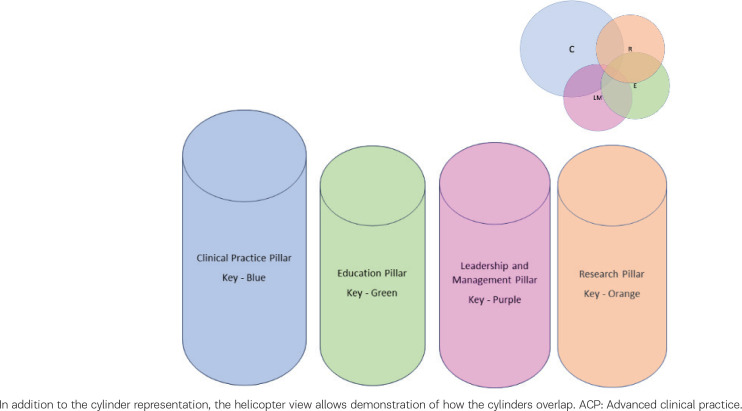
Composite case example of an ACP midwife, which demonstrates the weighting of involvement across the 4 pillars in this role

The ACP midwife is able to blend elements of the four pillars across the scope of their practice, at times overlapping and informing their development and expertise. These four pillars of advanced practice are represented visually as cylinders with two separate ‘viewpoints’: one viewing the cylinder straight on (Figure 1) and one from a helicopter view (Figure 2). The helicopter view allows demonstration of how the cylinders overlap, creating bubbles. Some practitioners had larger research or clinical practice bubbles, and smaller education or leadership. Activities such as leading a clinical research project would overlap between the clinical and research bubble, which this pictorial depiction clearly demonstrates. Advanced practice is about level of expertise, rather than formal title^2^, and this model allows midwives holding a variety of positions: specialist midwife, consultant midwife, research midwife, midwifery lecturer – to consider how they would map their qualities and experience against the framework of advanced practice to identify areas for development and career progression.

Folliard^15^ discusses defining the clinical academic midwife as a matter of identity, and the same is true for the ACP. The bubbles exemplify the multiple spheres of work a midwife may engage in, often labelled as having ‘fingers in many pies’, working together to construct the ACP identity and level of practice.

The ideal ACP would have an equal weighting of experience and involvement across all four pillars: 1) Clinical Practice, 2) Leadership and Management, 3) Education, and 4) Research. Often in practice, the elements are dictated by the job role and function. However, functional job roles although requiring advanced level skills and knowledge are not always equally weighted.

**Figure 3:**
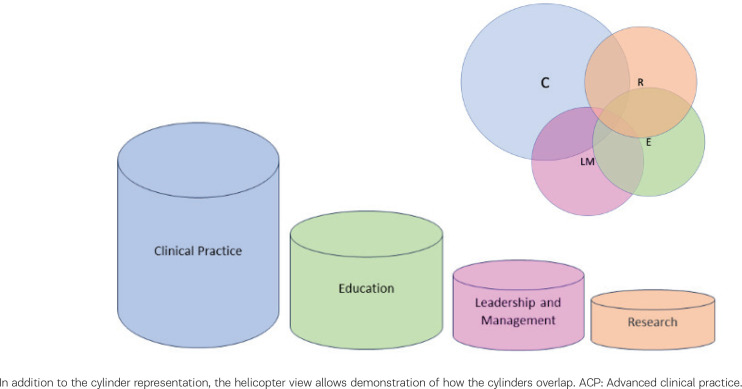
Composite case example of an ACP trainee, which demonstrates the developing areas of practice, with weighting of involvement across the 4 pillars in this midwifery role

A visual representation of this overlapping via the bubbles allows the identification of gaps for future development and may help with career conversations and training plans to gain a variety of clinical, educational and research expertise. This is demonstrated in the composite case study of an ACP Trainee (Figure 3).

## METHODS

The work was designed and conducted as a qualitative stakeholder analysis, and as such ethical approval was not sought as we were asking only about individuals’ own job roles. All participants who were interviewed were asked for verbal consent to use their information as part of the study reporting. All were willing to share their views on this basis.

Communications inviting expressions of interest from midwives working in NHS Trusts across England were circulated during June–July 2022 among midwifery professional networks and via Twitter and Instagram. A convenience sampling technique was used to gain representation from newly qualified preceptee midwives to consultant midwives. Although convenience sampling is subject to multiple forms of bias, it was deemed an appropriate method as it was cost-effective and allowed the team to draw upon existing professional networks. Steps were taken to improve credibility through recruiting as many participants as possible and identifying any possible external bias^16^.

In response, we received 54 expressions of interest and 31 midwives completed either a focus group or individual interview between July and September 2022. Among the 31 midwives there were consultant midwives, advanced midwife practitioners and trainees as well as midwives not yet on a training pathway aspiring towards advanced practice level (Table 1). Focus groups and one-to-one interviews were facilitated by the research team. A qualitative approach was taken, with a mix of focus groups and interviews chosen to maximize the opportunity for rich data reflective of individual and collective multifaceted experience^17^. Midwives are chronically understaffed and struggle to find time to dedicate to continuous professional development^18^. Therefore, it was a priority to maximize flexibility in recruiting participants to engage in this research. All the focus groups and interviews took place online via Microsoft Teams and were recorded.

**Table 1:** The professional role characteristics of the midwifery participants

*Stage of midwifery career*	*Number of participants*	*Range of experience at this stage of career in months/years*
Preceptee/early career (up to two years post-qualification)	7	10 months to 2 years
ACP trainees (imminently starting or already undertaking ACP pathway)	7	0–3 years on ACP training pathway
ACP midwives	7	1 month to 18 years as qualified ACP midwives
Consultant midwives	10	1 –8 years

ACP: Advanced clinical practice.

### Data collection and analysis

The focus group and interview participants were asked to discuss a range of questions related to their career experiences, with topic prompts adjusted according to the demographic of the group (Table 2). Within midwifery, there are hierarchical relationships amongst midwives at different stages within their careers^19,20^. To ensure all felt safe to share, we separated the groups by level of experience to encourage open expression. Contemporaneous interview notes were taken, and recordings were reviewed to identify additional material of relevance. The data were used to identify themes relating to career advice, education pathways, mentorship, and role modeling. Composite case studies were built to represent different career trajectories highlighting aspects of professional developmental and education. Each episode of data collection was undertaken by two members of the research team who subsequently analyzed the findings collaboratively to provide additional verification. This enabled the research team to take a holistic view of the data, analyzing the material reflexively, and acknowledging their positionality as midwives interviewing other midwives^21^. Following data collection, the research team reviewed the interview data with midwives at different stages in their careers. These were critically examined through rigorous conversation amongst the research team to reach consensus of overarching themes across the participant groups.

**Table 2:** Examples of questions used for each participant group (preceptor or newly qualified midwives, trainee or qualified ACP midwives, and consultant midwives) during focus groups and interviews between June and July 2022, from the data collection phase of stakeholder analysis (N=31)

***Preceptee midwives* (n=7)**	
3	Who has provided you with your main professional support since qualification?
5	Have you identified someone to advise/assist you with your development?
7	What additional education or experiences have you sought outside of your role?
13	Have you used the RCM career development framework?
***Trainee and qualified ACP midwives (*n=14)**	
3	What made you decide to take this career route?
7	What do you aspire to do next?
11	Do or will you have a joint post? If you do, how is this organized and what are the challenges and benefits?
12	Please explain how your role will fulfil the clinical pillar of ACP
25	What are your aspirations for the population which you serve?
***Consultant midwives* (n=10)**	
1	How long have you been a qualified Consultant Midwife?
3	What was your biggest driver to get to your current role?
4	Did you have a defined career pathway to follow?
5	Do you feel that in your role any of the ACP pillars are more pronounced/emphasized than others?

RCM: Royal College of Midwives. ACP: Advanced clinical practice.

## RESULTS

The research team collaboratively sought codes which were constructed around a central organizing concept to determine overarching themes relating to career aspirations and goals of midwives in relation to the ACP role. Using reflexive thematic analysis, final themes were confirmed through an active and creative process by the research team, which resulted in three main theme definitions: 1) Midwifery autonomy, 2) Desire for progression, and 3) Avenues of support.

### Midwifery autonomy

One theme of major significance was midwives wanting progression whilst retaining a woman-centered position. The vision of aspiring ACP midwives was described as becoming a ‘maxi midwife’, not a ‘mini doctor’, emphasizing the desire to retain the relational care with women and families. Midwives wanted to provide continuous, high-quality care, preserving the unique midwifery role and being able to stay with clients and lead care in collaboration with obstetric and other medical colleagues. The desire for autonomous clinical practice and control over their working environment can be unattainable within the hierarchy within which midwives operate, where medical clinical decision-making is viewed with greater seriousness than midwifery^22^.

Participants discussed the importance of remaining clinical throughout their career progression rather than reducing direct patient contact as they became more senior, enabling them to provide comprehensive advanced skills from an autonomous holistic position. This was exemplified by the clinical pillar being the most pronounced across the trainee and qualified ACP participants.

Many ACP midwives discussed how they enjoyed leadership but wanted this to be possible outside the managerial arena. Established ACP midwives reported their long-term goals were to build an advanced workforce which could provide holistic midwifery care to a variety of service users within specific populations. Through developing the ACP career pathway, it was deemed a positive way to inspire and motivate junior midwives and improve workforce retention.

There was a heavy emphasis on academic achievement and the importance of promoting holistic care in general maternity as well as specialist areas. Many participants had undertaken additional training within a variety of specialties during their formal ACP MSc programs, choosing modules which related to their own career aspirations. The majority of participants stated that prescribing was important in retaining workplace autonomy and ensuring midwifery care was a seamless experience for clients. Many consultant midwives reported that this role was still not recognized in wider midwifery circles, making their work frustrating. As a result, midwives felt like an anomaly within the midwifery team and ‘not part of the club’. This meant that midwives searched each other out in developing advanced roles across the country, tapping into networks of allied practitioners, with consultant midwives reporting that strategic work was often nationally focused rather than locally centered.

### Desire for progression

The aspiration for career progression was extremely high at each career stage with midwives taking a self-driven approach toward extending their existing skillset. Midwives expressed a clear determination in wanting to progress in their clinical role with service user contact. All participants stated that most of the visible career progression within midwifery was managerial, with many ACP trainees reporting that it was challenging to ‘do different’. Instead, they sought out opportunities to piece together a skill-set which exemplified their own midwifery passion and those which were seen as lacking within their own service. However, this was felt to be a lengthy and problematic approach to progression. Junior midwives were unclear how to progress, and to gain more experience in more complex care provision these newly qualified participants had ‘acted up’, trying out roles such as the practice development midwife, or managerial positions exploring progression for a finite time frame to see if this was something they wanted to pursue.

Midwives who held joint contracts as part of their advanced practice role with local Higher Education

Institutions felt this was a helpful gateway to inspire the next generation of midwives, demonstrating an alternative career pathway to that of a manager and to ensure that a range of opportunities were available once they had qualified. Newly qualified midwife (NQM) participants described frustration towards the need to demonstrate poorly defined ‘more experience’ before any further educational opportunities were offered. The perception was often that there was a lack of vision or support from senior colleagues. For some NQMs, this spurred them on to look for progression outside their employing organization, not because they did not like their daily work, but due to a lack of support.

Education was an important part of progression and as part of the consultant midwife role, some had established links with universities as associate tutors, were established as midwifery lecturers, and some had published research following completion of their MSc or PhD. It was acknowledged by midwives at all career stages that education was mostly self-funded, done in their own time, and self-driven. This was deemed necessary to develop their profession and enable the maternity provision to align with local population needs.

Both personal motivation and self-drive were identified as being the most significant facilitator for the career development. Each midwives’ journey to becoming an advanced practitioner was individual and took different routes with focus shifting as they encountered new learning opportunities and became more proficient in their advanced practice role. Many midwives had links to midwifery innovators and expressed significant levels of inspiration and aspiration to be an innovator and implement change to promote advocacy and holistic care for service users.

### Avenues of support

Participants discussed the need for role models in senior clinical positions as this is something they had lacked themselves. Many participants discussed finding support and role models outside their immediate midwifery colleagues, including obstetricians, consultant midwives, and ACP professionals from other areas. Some directors or heads of midwifery had a clear vision which included the ACPiM role, but this was challenging to fund and secure. The changing nature of leadership within maternity made this more challenging, and conflict was present between job descriptions for consultant roles and the vision of the director or head of midwifery.

Established ACP and consultant midwives felt as though they were often on their own because ‘top-down working’ made it more challenging to forge professional relationships. It was felt that they could make the most difference when senior support aligned with the service vision. Participants wanted to enable collaborative innovation, and many voiced their aspirations to act as mentors for aspiring ACP midwives. Some consultant midwives felt it was important that career progression should not include solely managerial attainment but advancing clinical skills, particularly for those aspiring to be advanced clinical practitioners.

NQMs had varying degrees of support, with no participants describing a named mentor or preceptor midwife. There were a variety of external networks which they individually utilized to seek out support and development opportunities. Most of the consultant midwives interviewed reported that many of these working relationships and collaborative working groups had formed more by organically by chance than by design. These themes created a deeper understanding of the resulting barriers and facilitators to progression.

Although the scope of the research did not include the enhanced practitioner, it was acknowledged across the stakeholder group that an enhanced or specialist role was often a first step to advancing one’s practice. Although none of the participants interviewed was classified as ‘enhanced practitioner’, some ACP trainees were perceived by others (and themselves) as more ‘enhanced practitioners’ than ACP trainees. This indicates the need for clearer definitions across these roles regarding career progression^13^.

## DISCUSSION

One significant finding from the primary data collection was the importance of the barriers and facilitators experienced for individuals to progress within their midwifery careers. These pertained across personal and professional development, workforce education and training plans and can be understood within the cultural, organizational, and personal context of midwives’ experiences. Many of the stakeholders involved in the research discussed the spread of their ACP weighting as not evenly distributed across the four pillars, instead being closely aligned to the everyday practice in their role (Figures 1 and 2). To fully comprehend the variety of pathways for midwives to progress in their careers it is important to consider those factors which work against them, and in support of them.

### Cultural factors

Cultural factors have been deemed significant in affecting patient safety and staff retention^18,23^. Little research has commented on the role of workplace culture in midwifery career development. The midwives interviewed for this research expressed that workplace culture was hugely significant in developing a midwife’s sense of self, their ambition, and opportunities to progress.

The NQM participants valued a need to feel grounded as registered midwives above anything else. They were pre-occupied with ‘surviving’ rather than considering long-term career plans. This is understandable given that the majority of newly qualified midwives leave the NHS within a year post-qualification due to a variety of factors including workload, staffing, and burnout^18^. Where NQMs were offered training events, they were often pulled to work clinically and missed out on these developmental opportunities. These training sessions were not rearranged, the priority being to cover clinical acuity.

The UK WHELM study found that a culture lacking opportunities for professional development was largely associated with burnout, depression, and anxiety^24^. Midwives wanted to progress, but a lack of support made them feel disempowered. A report into workforce bullying within midwifery found that midwives are consistently disempowered, with institutional cultures meaning there are negative repercussions for questioning hierarchy and ‘speaking out’^20^. Midwives work in an atmosphere where they may be ignored when raising clinical safety issues, feel unsupported and undervalued. It is therefore understandable how midwives may feel the need to ‘pick their battles’ in terms of which issues they decide to raise with management teams, their own personal ambition feeling less important than pressing clinical safety issues. Safety reports highlight how incivility, bullying and human factors have significant implications for patient safety^23,25^. Workplace culture may also act as a barrier for staff development, staff satisfaction and therefore retention.

This study found that throughout midwives’ careers there was a lack of ‘career conversations’ or support for development. Junior midwives were informed they would not be supported to engage in further study until they had been qualified for three years. Due to the dearth of ACP role models, trainee ACPs did not know how to access midwifery support for ACPiM role development, instead seeking mentorship from obstetricians. It was noted by participants that obstetricians may be more supportive of further training beyond qualification, as this is the way in which medical careers are shaped.

### Organizational systems

Growing, maintaining and supporting the future midwifery workforce has been recently highlighted as one of the four main themes of the NHS England three-year delivery plan for maternity and neonatal services^26^. This report highlights the need for a safer, more personalized and equitable service, comprising highly skilled individuals. However, it also draws attention to the enormous pressures that existing staff face, and the importance of maintaining opportunities for staff progression within this context. Midwives participating in this study, stated they experienced a lack of clear service vision from management, with frequent changes of leadership cited as a reason that progress in developing the service was a challenge. A shared global service vision has been noted as a key mechanism in improving midwifery workplace culture^27^.

There was also concern expressed among participants regarding lack of funding or protected time to complete ACP training, limited job descriptions, and an expectation to cover understaffed clinical areas within the department, factors which were identified as barriers across all career stages. Avery et al.^28^ observed that among nurses, midwives and allied health professionals (NMAHPS) wishing to pursue clinical academic careers, lack of clear career paths and funding for these combined roles, hindered progression. This is also seen in midwives’ ability to secure funding following Master’s level training, an issue identified by funding bodies such as the National Institute of Health Research^29-31^. Frequent changes in management, lack of protected time and funding, as well as staffing issues, mean that midwives are often needed to work in patient facing roles on the ‘shop floor’^20^.

Participants reported role confusion among their midwifery colleagues and leadership teams; however, their role was better understood by nursing ACP colleagues and the obstetric teams. A lack of clarity and understanding about the nature of the ACP nurse role has equally been a concern, despite attempts to conceptualize and progress advanced nursing practice^32^, suggesting that work to gain clarity among the maternity workforce, which this research contributes to, will be ongoing.

A focus on sustaining the service, rather than developing the individual, emerged as a concern for participants in this study. For some, this was experienced as training opportunities being offered to fill specific roles that happened to be empty. For example, some were encouraged to undertake training in leadership and management, rather than being offered a breadth of opportunities across the four pillars or in a direction of personal and professional interest to them. This focus on staffing the service rather than meeting the population needs through service development may be a key barrier to ACP development in maternity care. The importance of a shift in healthcare service culture that enables training opportunities in research for NMAHPS to be viewed as a transformative rather than transactional act, and therefore optimizes NMAHP personal development, is advocated^33^.

### Strengths and limitations

The strength of this work was the range of midwives’ views that were present, from all career stages. It was a limitation that this was a self-selecting group and the opportunity to join the study was available for a short period of time, so some potential participants may have been missed.

## CONCLUSIONS

This study highlights that midwives have a strong desire to continually develop expertise, but this needs support at an institutional level and embedding in the wider culture of midwifery practice. This opportunity should exist from the point of registration and continue throughout the midwife’s career. This article has highlighted multiple barriers and facilitators for midwives to progress on an ACP pathway. Awareness of these cultural and organizational factors and the ability to address them should be available for midwives. The onus for this cannot be solely the responsibility of the individual, but commitment and organizational change and support is needed alongside the role of the ACP midwife being valued within the multidisciplinary team and wider NHS. Ultimately the benefits of this will not only be felt by individuals but within broader healthcare provision in supporting recruitment and retention, and a level of midwifery practice which promotes improved outcomes for families.

## Data Availability

The data supporting this research are available from the authors on reasonable request.
